# Day–night pattern of energy expenditure and body temperature in cachectic tumour-bearing rats

**DOI:** 10.1054/bjoc.1999.1469

**Published:** 2000-10

**Authors:** H Oudart, A Malan, Y le Maho, A Geloen

**Affiliations:** Centre d’Ecologie et Physiologie Energétiques, associé à l’Université Louis Pasteur, CNRS, 23 rue Becquerel, Strasbourg, F-67087, France; Thermorégulation et Energétique de l’Exercice, Université Lyon I, 8 avenue Rockfeller, Lyon, Cedex 08, F-69373, France

**Keywords:** Yoshida sarcoma, energy metabolism, indirect calorimetry, cancer cachexia

## Abstract

The implication of an increase in energy expenditure in cancer cachexia, which seems to be related to the type of tumour, remains unclear. We therefore investigated the energy metabolism and body temperature in anorectic and cachectic rats bearing the Yoshida sarcoma (TB), in comparison with pair-fed (PF) and ad-libitum fed (AL) control rats. The resting energy expenditure was higher in the TB than in the two control groups when corrected for the modifications of body composition. However, the total energy expenditure did not differ between the TB and the AL, presumably because of the drop of activity in TB. There was a temporal distribution of differences in energy expenditure with higher energy expenditure in TB than in AL during the diurnal phase and a lack of difference during the nocturnal phase. The TB presented a fever, which was limited to the diurnal period. Moreover, the acrophase of the body temperature rhythm was delayed in the TB. These results highlight the circadian effects of tumour development on the energy metabolism of the host and hint to the possible implication of cytokines. © 2000 Harcourt Publishers Ltd © 2000 Cancer Research Campaign


British Journal of Cancer (2000) 83(8), 1055–1060
© 2000 Cancer Research Campaign
doi: 10.1054/ bjoc.1999.1469, available online at http://www.idealibrary.com on IDEAL®

### Day–night pattern of energy expenditure and body temperature in cachectic tumour-bearing rats

**H Oudart[1], A Malan[1], Y le Maho[1] and A Geloen[2]**

[1]Centre d'Ecologie et Physiologie Energétiques, associé à l'Université Louis Pasteur, CNRS, 23 rue Becquerel, F-67087 Strasbourg, France; [2]Thermorégulation et Energétique de l'Exercice, Université Lyon I, 8 avenue Rockfeller, F-69373 Lyon, Cedex 08, France

**Summary** The implication of an increase in energy expenditure in cancer cachexia, which seems to be related to the type of tumour, remains unclear. We therefore investigated the energy metabolism and body temperature in anorectic and cachectic rats bearing the Yoshida sarcoma (TB), in comparison with pair-fed (PF) and ad-libitum fed (AL) control rats. The resting energy expenditure was higher in the TB than in the two control groups when corrected for the modifications of body composition. However, the total energy expenditure did not differ between the TB and the AL, presumably because of the drop of activity in TB. There was a temporal distribution of differences in energy expenditure with higher energy expenditure in TB than in AL during the diurnal phase and a lack of difference during the nocturnal phase. The TB presented a fever, which was limited to the diurnal period. Moreover, the acrophase of the body temperature rhythm was delayed in the TB. These results highlight the circadian effects of tumour development on the energy metabolism of the host and hint to the possible implication of cytokines.
© 2000 Harcourt Publishers Ltd

**Keywords**: Yoshida sarcoma; energy metabolism; indirect calorimetry; cancer cachexia

Tumour-bearing is frequently associated with a deterioration of the body condition of the host, resulting in cancer cachexia. This syndrome is characterized by a loss of body lipid and proteins (Giacosa et al, 1996) and an increase in body water content (Rechcigl et al, 1961). Anorexia is a common feature of cancer cachexia, whereas the level of energy expenditure of the host has been shown to be either increased, decreased or unchanged (Keller, 1993). This effect on energy expenditure may be in relation to the type of tumour (Fredrix et al, 1991).

In healthy humans and animals, the level of energy expenditure is directly related to the fat-free dry mass (Cunningham, 1991), i.e. the metabolic active tissues which determine the level of the resting energy expenditure. The other components of the total energy expenditure are thermogenesis, activity energy expenditure and diet-induced thermogenesis. During cancer cachexia, an increase in the level of energy expenditure can be observed despite a loss of fat-free dry mass of the host. In this way, in cancerous patients, the resting and the non-resting energy expenditure could contribute to elevation of the energy expenditure (Warnold et al, 1978; Lindmark et al, 1984). In a rat model of cancer cachexia using the Morris hepatoma, the resting energy expenditure is increased, whereas the total energy expenditure is unchanged, due to a decrease in activity thermogenesis (Luketich et al, 1990).

Several studies have indicated an association between cancer cachexia and fever (McCarthy and Daun, 1993; Tessitore et al, 1993). This increase in body temperature could be, at least in part, responsible for an increase in energy expenditure. However, some models of experimental cancer cachexia lead to a decrease of body temperature (Smith et al, 1993) or to a fever in the first stage of tumour development followed by a decrease under the normal value in the later stage (Tessitore et al, 1993).

Thus, we investigated here the energy expenditure and the body temperature in a well-characterized model of cancer cachexia. We used the Yoshida sarcoma which induces an anorexia, a wasting of muscle protein (Temparis et al, 1994), a loss of body lipid and protein, and a negative nitrogen balance in the rat (Oudart et al, 1993). The energy expenditure was measured by indirect calorimetry during the last 24 h of tumour development and the results were standardized in relation to the modifications of body composition. The body temperature was measured throughout the tumour development.

### MATERIALS AND METHODS

### Animals, tumour and tumour transplantation

Male Wistar rats were purchased from IFFA-CREDO (Lyon, France). They were kept in individual cages and provided ad libitum with water and a standard diet (A03, UAR, France). Room temperature was 26 ± 1°C. Light was on from 08:00 h to 20:00 h. After 1 week, the tumour was implanted (TB group). Tumour was implanted intramuscularly (i.m.) in the left rear leg with a small piece of Yoshida sarcoma (1 $mm^3$). Control rats were sham-operated and received a heat-killed (60°C, 30 min) piece of tumour. One group of control rats was pair-fed to the ad libitum food intake of their respective tumour-bearing (PF group). Pair-feeding was achieved by giving each pair-fed control the previous day intake of its respective tumour-bearing rat. Ten days after tumour implantation, which is a duration of tumour development that avoids ulceration (Oudart et al, 1993), tumour-bearing and ad libitum-fed control rats (AL group) were killed by cervical dislocation between 9:00 and 10:00 h. Corresponding pair-fed controls were killed 1 day later, according to the pair-feeding pattern. The tumour, the retroperitoneal and epididymal white fat pads, the

*Received 14 October 1998*
*Revised 20 April 1999*
*Accepted 20 April 1999*

*Correspondence to:* H Oudart

**Table 1** Body mass, body composition, and organ masses in the tumour-bearing rats (TB), pair-fed control rats (PF), and adlibitum-fed control rats (AL)

| | TB | PF | AL |
|---|---|---|---|
| Initial body mass, g | 200.5 ± 8.2 | 201.8 ± 3.5 | 202.3 ± 3.5 |
| Tumour mass, g | 14.5 ± 1.3 | – | – |
| Final carcass mass, g | 230.6 ± 3.5[a] | 225.6 ± 4.8[a] | 263.8 ± 1.5 |
| Carcass mass gain, g | 30.6 ± 3.2[a] | 23.5 ± 3.0[a] | 62.1 ± 4.1 |
| LBM, g | 231.1 ± 4.6[a,b] | 211.3 ± 5.6[a] | 247.3 ± 2.8 |
| FFDM, g | 59.2 ± 4.4[a] | 57.6 ± 3.6[a] | 68.4 ± 1.9 |
| EPI, g | 1.53 ± 0.09 | 1.58 ± 0.13 | 1.76 ± 0.11 |
| RP, g | 0.98 ± 0.10[a] | 1.16 ± 0.14 | 1.47 ± 0.13 |
| Soleus, mg | 142.2 ± 10.9[a,b] | 156.5 ± 13.5 | 156.2 ± 15.7 |
| EDL, mg | 106.6 ± 3.1[a,b] | 114.3 ± 3.7 | 118.5 ± 3.0 |
| Liver, g | 11.7 ± 0.4[b] | 9.7 ± 0.3[a] | 12.2 ± 0.4 |
| BAT, mg | 197.8 ± 10.1[a,b] | 247.2 ± 16.0 | 299.7 ± 20.4 |

LBM: lean body mass including tumour mass; FFDM: fat-free dry mass; EPI: epididymal white fat pads; RP: retroperitoneal white fat pads; EDL: extensor digitorum longus; BAT: brown adipose tissue. [a]$P < 0.05$ vs AL; [b]$P < 0.05$ vs PF. $n = 14$ per group. Means ± s.e.m.

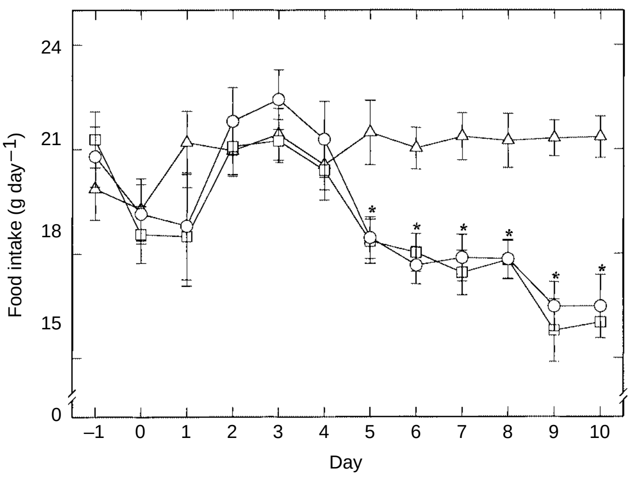

**Figure 1** Food intake of tumour-bearing rats (○), pair-fed control rats (□), and ad libitum-fed control rats (△) during the tumour development. Day 0: day of the implantation of the tumour. *$P < 0.05$ vs ad libitum-fed control rats; $n = 14$ per group. Means ± s.e.m.

interscapular brown adipose tissue, the liver, and the soleus and extensor digitorum longus muscles were rapidly excised and weighed. The carcasses (body minus tumour) were frozen in liquid nitrogen and stored at –20°C until composition analysis.

All experiments were done in compliance with European Union regulations on care of experimental animals, and submitted to control by French authorities.

### First experiment: measurement of energy expenditure

In 18 rats (six in each group) oxygen consumption was measured with an open-circuit indirect calorimetry (Klogor, France), as previously described (Cimmino et al, 1996). Briefly, oxygen concentration of the outgoing air was successively measured in five different cages. The system was rinsed during 90 s between each cage measurement. The final value for oxygen concentration of the outgoing air was the mean of 10 measures obtained during 40 s. Each cage was sampled every 11 min, one cage was left vacant and was used as reference for measuring ambient oxygen concentration. Oxygen concentration was measured continuously over 23.5 h per day, a 30-min period being required for calibration of the oxygen analyser. One hundred and twenty-seven measures were collected per day for each rat. The average of the five lowest values was considered as corresponding to resting energy expenditure. Energy expenditure was obtained by using an energy equivalent of 20.27 J $ml^{-1}$ $O_2$.

Oxygen consumption was measured during the days 9 and 10 of post-implantation (the two last days before the slaughtering of the animals). Day 9 was considered as acclimatization and only day 10 was considered for the determination of energy expenditure.

### Second experiment: measurement of body temperature

Battery-operated biotelemetry transmitters (Aktiv 500 Telemetry System, GFT, Kiel, Germany) were implanted intraperitoneally in 24 rats. Body temperature was recorded every 9 min with an automatic data collection system. One week later, tumour was implanted in eight rats (TB group), heat-killed tumour was implanted in eight other rats (AL group). The following day, heat-killed tumour was implanted in the last eight rats (PF group).

The data of body temperature were analysed using the equation:

$$y = [A + B\cdot\cos(2\pi\cdot(t\text{–}C)/24)]$$

where A was the mean daily body temperature (°C), B the amplitude of body temperature peak (°C), C the time of acrophase (h), and t the standard time (h). This cosinor analysis was performed on consecutive 4-day periods; each period of analysis began 2 days before the end of the previous period of analysis.

### Body composition analysis

For body composition analysis, the carcasses (bodies minus tumour) were ground under liquid nitrogen, lyophilized, and ground again to a fine powder. Total lipid was determined gravimetrically by a method adapted from Folch et al (1957). Lean body masses (LBM) were determined by subtracting fat masses from body masses. Fat-free dry masses (FFDM) were determined by subtracting water masses from lean body masses. LBM and FFDM included the tumour mass in the TB group.

### Statistical analysis

Results are expressed as the means ± s.e.m. Statistical comparisons were performed with one-way ANOVA, followed by Tukey test for multiple comparison or with Kruskall–Wallis test when appropriate. Statistical significance was set at $P < 0.05$.

The data of food intake, body mass, body composition and organ masses were found to be similar in the two experiments. The results were therefore pooled and are presented as a single table.

### RESULTS

### Food intake, body and organ masses

Food intake (Figure 1) and body mass (Table 1) were similar in the three groups of rats at the beginning of the experiment. In

 *© 1999 Cancer Research Campaign*

**Table 2** Energy expenditure in the tumour-bearing rats (TB), pair-fed control rats (PF), and ad libitum-fed control rats (AL)

| | TB | PF | AL |
|---|---|---|---|
| Total energy expenditure | | | |
| W | 1.56 ± 0.07[c] | 1.38 ± 0.06[a] | 1.68 ± 0.04 |
| W/kg LBM | 6.74 ± 0.19 | 6.52 ± 0.16 | 6.80 ± 0.15 |
| W/kg FFDM | 26.8 ± 0.8[c] | 24.0 ± 0.9 | 24.7 ± 0.7 |
| Nocturnal energy expenditure | | | |
| W | 1.70 ± 0.09[b] | 1.52 ± 0.09[b] | 1.96 ± 0.06 |
| W/kg LBM | 7.34 ± 0.27 | 7.16 ± 0.23 | 7.92 ± 0.22 |
| W/kg FFDM | 28.9 ± 1.0 | 26.3 ± 1.2 | 28.6 ± 0.9 |
| Diurnal energy expenditure | | | |
| W | 1.41 ± 0.05[d] | 1.24 ± 0.04[a] | 1.40 ± 0.03 |
| W/kg LBM | 6.10 ± 0.14[b] | 5.86 ± 0.12 | 5.66 ± 0.11 |
| W/kg FFDM | 25.0 ± 0.6[a,c] | 21.5 ± 0.6 | 20.8 ± 0.4 |
| Nocturnal resting energy expenditure | | | |
| W | 1.32 ± 0.05[b,d] | 1.06 ± 0.05[b] | 1.48 ± 0.04 |
| W/kg LBM | 5.73 ± 0.18[d] | 5.02 ± 0.14[b] | 5.99 ± 0.17 |
| W/kg FFDM | 22.9 ± 0.8[d] | 18.4 ± 0.7[b] | 21.6 ± 0.7 |
| Daily resting energy expenditure | | | |
| W | 1.16 ± 0.04[d] | 0.97 ± 0.04[b] | 1.15 ± 0.02 |
| W/kg LBM | 5.03 ± 0.13[a,d] | 4.56 ± 0.11 | 4.65 ± 0.10 |
| W/kg FFDM | 20.6 ± 0.7[a,c] | 16.8 ± 0.5 | 17.1 ± 0.3 |

LBM: lean body mass; FFDM: fat-free dry mass. [a]*P* < 0.05, [b]*P* < 0.01 vs AL; [c]*P* < 0.05, [d]*P* < 0.01 vs PF. *n* = 6 per group. Means ± s.e.m.

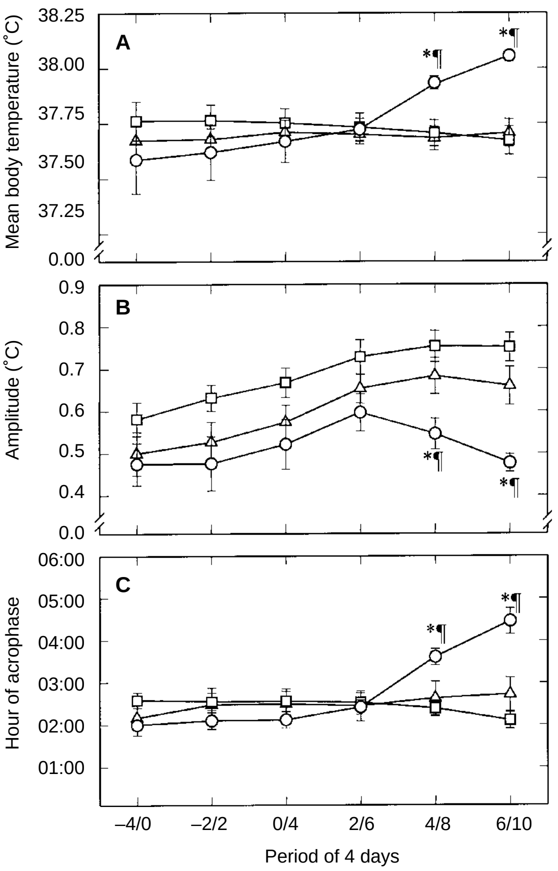

**Figure 2** Mean body temperature (**A**), amplitude of the body temperature rhythm (**B**), and hour of the acrophase (**C**) in tumour-bearing rats (○), pair-fed control rats (□) and ad libitum-fed control rats (△) determined by the cosinor method on periods of four nycthemerons, each period of determination beginning 2 days before the end of the preceding period (for full details, see the Materials and Methods section). *$P$ < 0.05 vs ad libitum-fed control rats; ¶$P$ < 0.05 vs pair-fed control rats. $n$ = 8 per group. Means ± s.e.m.

tumour-bearing rats (TB), food intake decreased 5 days after implantation of the tumour. Their food intake was compared to the food intake 1 day before the tumour implantation, i.e. baseline value, and to the ad libitum fed control rats (AL). After 10 days of tumour growth, the food intake of the TB group was about 77% of the baseline value and of the food intake of the AL group (Figure 1). During the period of tumour growth, i.e. 10 days, the pair-feeding ensured an equivalent energy intake between the TB group and the pair-fed group (PF) (Figure 1).

The final carcass mass (body mass minus tumour) and the carcass mass gain did not differ between the TB group and the PF group and were decreased in these two groups compared to the values of the AL group (Table 1). The LBM including the tumour mass was higher in the TB rats than in the PF ones. However, this difference vanished when the tumour mass was not included (216.6 ± 3.9 g in TB rats vs 211.3 ± 5.6 g in PF rats). The FFDM, similar in the TB and PF groups, was lower than in the AL group (Table 1).

The mass of epididymal white fat pads was similar in the three groups. The mass of retroperitoneal white fat pad was lower in the TB and PF groups than in the AL group but only the difference between TB and AL groups reached the statistical significance (Table 1). The masses of muscles (EDL and soleus) and BAT were decreased in the TB group compared to the two control groups. The mass of liver was lower in the PF group than in the TB and AL groups.

### Energy expenditure

Expressed in Watts (W) (irrespective of body mass), the total energy expenditure in the PF group was 19% lower than in the AL group and 11% lower than in the TB group. The total energy expenditure did not differ between the TB and AL groups (Table 2). Whatever the period (diurnal, nocturnal), the energy expenditure in the PF group was lower than in the AL group. Likewise, the resting energy expenditure was 16% lower in the PF group than in the AL group. The diurnal and the nocturnal resting energy expenditure were respectively 13% and 11% lower in the TB group than in the AL group. The diurnal, nocturnal resting and resting energy expenditure were respectively 12%, 20%, and 16% higher in the TB group than in the PF group (Table 2).

Expressed per unit of LBM, the total energy expenditure did not differ between the three groups. Excepted for the nocturnal resting energy expenditure, there was no difference between the PF group and the AL group. The diurnal and resting energy expenditures were 7% higher in the TB group than in the AL group. Likewise, the nocturnal resting and the total resting energy expenditure were, respectively, 12% and 9% higher in the TB group than in the PF group (Table 2).

Expressed per unit of FFDM, the results were similar to the results expressed per unit of LBM, excepted for the total and the diurnal energy expenditures. In the TB group, the total and the diurnal energy expenditures were respectively 12% and 16% higher than in the PF one (Table 2).

© 1999 Cancer Research Campaign

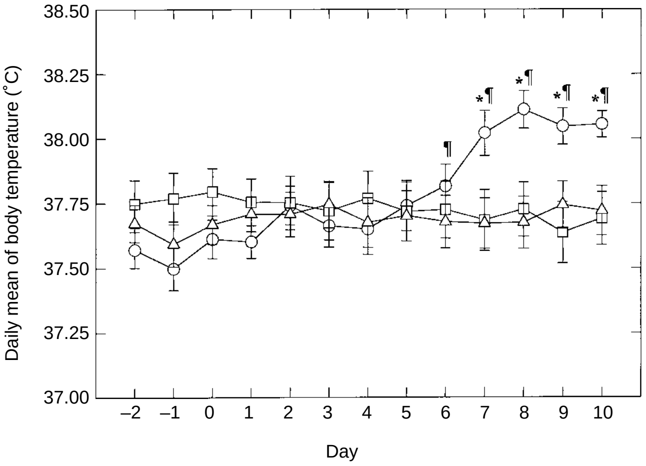

**Figure 3** Daily mean body temperature in tumour-bearing rats (○), pair-fed control rats (□), and ad libitum-fed control rats (Δ) during the tumour development. Day 0: day of the implantation of the tumour. **P* < 0.05 vs ad libitum-fed and pair-fed control rats; ¶*P* < 0.05 vs the day preceding the day 0. *n* = 8 per group. Means ± s.e.m.

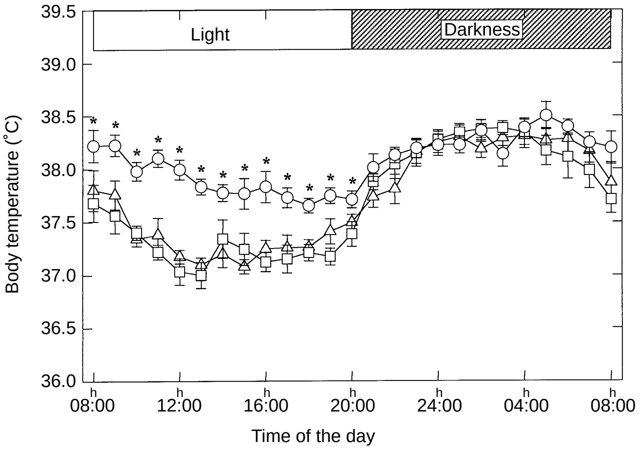

**Figure 4** Hourly mean body temperature in tumour-bearing rats (○), pair-fed control rats (□), and ad libitum-fed control rats (Δ) during the last nycthemeron of tumour development. **P* < 0.05 vs ad libitum-fed and pair-fed control rats. *n* = 8 per group. Means ± s.e.m.

### Body temperature

Mean body temperature as determined by cosinor analysis (Figure 2) was 0.35°C higher in the TB group than in the two control groups during the period from day 6 to day 10 after tumour implantation (Figure 2A). The amplitude of the body temperature rhythm was 0.25°C lower in the TB group than in the two control groups (Figure 2B). The time of acrophase of body temperature rhythm was delayed in the TB group compared to the PF and AL groups. During the days 6–10 after tumour implantation, the delay was about 2 h (Figure 2C).

The increase in daily mean body temperature started the 6th day post-implantation. The daily mean body temperature was significantly elevated in the TB group from the 7th day and was 0.35°C higher in the TB group than in the two control groups during the last 3 days of tumour growth (Figure 3). Moreover, the higher body temperature in the TB group than in the two control groups was limited to the light period of the nycthemeron (Figure 4).

### DISCUSSION

The results of the studies concerning the involvement of elevated energy expenditure in the aetiology of cancer cachexia are contradictory. Studies report either an increase, a decrease, or no change in the energy expenditure of cancerous humans and animals (Keller, 1993; Ogilvie et al, 1993). Such conflicting results might be explained by different tumour types (Fredrix et al, 1991).

Our study was undertaken to investigate the modification of energy metabolism in rats bearing the Yoshida sarcoma. This tumour has a rapid development and induces cachexia characterized by anorexia, a degradation of nitrogen balance, and an alteration of the host body composition (Oudart et al, 1993). To our knowledge, our study is the first to give detailed information on the energetic changes related to the development of Yoshida sarcoma. Importantly, it gives for the first time results on the effects of tumour development on circadian aspects of energy expenditure and body temperature.

The Yoshida sarcoma bearing rats were anorectic and it is well known that anorexia is a common feature of tumour development. We did not determine the body composition in this study. However, the lower masses of retroperitoneal white fat pads and of muscles in the TB rats than in the control animals indicate that the Yoshida sarcoma induces a cachexia, as previously showed (Oudart et al, 1993). Moreover, the masses of muscles, retroperitoneal white fat pads and brown adipose tissue were lower in the TB group than in the PF group, implying that the cachexia induced by the Yoshida sarcoma is not solely due to the reduction of the food-intake. Such results are in accordance with a study on female rats which showed that the masses of gastrocnemius muscle and parametrial white fad pads are lower in Yoshida sarcoma bearing animals than in pair-fed control animals (McCarthy et al, 1993).

### Energy expenditure and body temperature: overall data

As demonstrated by the larger final than initial body masses, the energy balance remained positive in the TB and PF groups. However, the classical decrease in energy expenditure, which is associated with a decrease in food intake (Forsum et al, 1981) was observed in the PF group but not in the TB one. The total energy expenditure, excepted when expressed in W per unit of lean body mass, was higher in the TB rats than in the PF rats, indicating that an increase in the total energy expenditure is involved in the aetiology of the cachexia induced by the Yoshida sarcoma. The lack of difference in energy expenditure expressed in W per unit of lean body mass between the TB and PF rats was probably due to the increase in water body content which is observed in tumour-bearing rats (Oudart et al, 1993). So, to avoid the influence of the variations in body water, the better standardization of energy expenditure are in relation to the FFDM. In this way, the higher total energy expenditure in TB rats than in the PF rats evidences that the difference are not due to modifications of the body composition. Whatever the energy expenditure component (diurnal, nocturnal, resting), the value of the PF group was lower than the value of the AL group. These differences vanish when the components of the energy expenditure are expressed by unit of FFDM (excepted for the nocturnal resting energy expenditure), evidencing that the differences in energy expenditure between the

© 1999 Cancer Research Campaign

PF and AL groups were mainly due to the modification of body composition.

Expressed in watts per animal, the differences in the resting energy expenditure paralleled the differences observed in the total energy expenditure. However, contrary to the total energy expenditure, the resting energy expenditure expressed per unit of FFDM was higher in the TB group than in the two control groups, demonstrating that the Yoshida sarcoma induces an increase in the resting energy expenditure, despite the anorexia associated with the tumour development. Two hypothesis, not mutually exclusive, might explain the difference in resting energy expenditure, whereas the total energy expenditure did not differ between the TB group and the two control groups. First, the locomotor activity was dramatically decreased in Yoshida sarcoma-bearing rats compared to control animals (personal data). This lower physical activity in the TB rats than in control animals could at least in part explain the lack of difference in total energy expenditure, despite a higher resting energy expenditure in TB rats. Second, the food intake was lower in TB rats than in the AL rats. Thus it is highly probable that the energy expenditure due to feeding (diet-induced thermogenesis, at least the obligatory component) is lower in TB rats than in AL rats. This difference in diet-induced thermogenesis might also explain the observed results. Such decrease in activity and diet-induced energy expenditures leading to a lack of difference in total energy expenditure whereas the resting energy expenditure is elevated has been shown in rats bearing the Morris hepatoma (Luketich et al, 1990).

Fever is often associated with cancer cachexia (McCarthy and Daun, 1993; Tessitore et al, 1993), although cachexia induced by a tumour such as the methylcholantrene sarcoma is associated with a drop of body temperature (Smith et al, 1993). Rats bearing the Yoshida hepatoma, which is a tumour close to the Yoshida sarcoma, show an increase in body temperature during the first days of tumour development followed by a drop of body temperature below normal values in later stages (day 10 after tumour implantation) of tumour development (Tessitore et al, 1993). In our model of cancer cachexia, the tumour-bearing rats presented an increase in body temperature, which persisted until the last day of tumour development.

Fever associated with cancer cachexia or induced by endotoxin is due to the pyrogen effect of cytokines such as interleukin 1β and interleukin 6 (Busbridge et al, 1993; Roth et al, 1993), whereas tumour necrosis factor seems to attenuate the fever induced by the interleukin 1β or endotoxin (Long et al, 1992). There are no available data concerning cytokines in Yoshida sarcoma-bearing rats and we did not measure the circulating cytokines in the present study. Yet, it is possible that the Yoshida sarcoma induces high levels of circulating of cytokines in the host.

### Energy expenditure and body temperature: nycthemeral data

There are few reports of circadian rhythm of body temperature in tumour-bearing rats. To our knowledge, the most complete study was performed on rats bearing the methylcholantrene sarcoma, which induces a drop in body temperature (Smith et al, 1993). This drop is limited to the dark phase of the nycthemeron in the first stages of tumour growth and extended over the complete nycthemeron with the tumour development. In the Walker 256 carcinoma bearing rats, a fever is present throughout the nycthemeron (McCarthy and Daun, 1993). In our model, the fever was limited to the light phase of the nycthemeron throughout the tumour development. Moreover, the rats bearing the Yoshida sarcoma presented a delay in the acrophase of body temperature rhythm. To our knowledge, it is the first time that a phase shift in the body temperature rhythm is reported in tumour-bearing rats exposed to normal photoperiod.

In accordance with these changes in body temperature, the energy expenditure of tumour-bearing rats did not differ from controls during the dark phase of the nycthemeron whereas during the light phase, which is the period of lower physical activity in the rat, the energy expenditure was higher in tumour-bearing rats than in control rats.

This diurnal fever could be explained by the rhythm of cytokines production. Indeed, in human patients with colorectal or gastrointestinal cancers, a rhythm in circulating tumour necrosis factor is detected with a peak in the middle of the night (about 02:00 h) and a lower value in the afternoon (about 12:00 h) (Muc-Wierzgon et al, 1996, 1998). The effect of cytokines may also be subjected to day–night modulation. Indeed, it has been shown in mice that interferon α induces fever during the light phase but not during the dark phase of the nycthemeron (Ohdo et al, 1997).

Our study therefore demonstrates that the Yoshida sarcoma induces an increase in resting energy expenditure and a fever, which are limited to the light phase of the nycthemeron. Moreover, there is a phase shift in the circadian rhythm of body temperature. Cytokines are probably implicated in this effect. Further experiments should be performed to determine the role of the different cytokines in the fever and in the increase in energy expenditure, and particularly in the circadian aspects.

### ACKNOWLEDGEMENTS

This work was supported by a grant from the Association pour la Recherche sur le Cancer.

#### REFERENCES

Busbridge NJ, Dascombe MJ and Rothwell NJ (1993) Chronic effects of interleukin-1-beta on fever, oxygen consumption and food intake in the rat. *Horm Metab Res* **25**: 222–227

Cimmino M, Mion F, Goglia F, Minaire Y and Geloen A (1996) Demonstration of in vivo metabolic effects of 3,5-diiodothyronine. *J Endocrinol* **149**: 319–325

Cunningham JJ (1991) Body composition as a determinant of energy expenditure: a synthetic review and a proposed general prediction equation. *Am J Clin Nutr* **54**: 963–969

Folch J, Lees M and Stanley GHS (1957) A simple method for the isolation and purification of total lipids from animal tissues. *J Biol Chem* **226**: 497–509

Forsum E, Hillman PE and Nesheim MC (1981) Effect of energy restriction on total heat production, basal metabolic rate, and specific dynamic action of food in rats. *J Nutr* **111**: 1691–1697

Fredrix EWHM, Soeters PB, Wouters EFM, Deerenberg IM, Von Meyenfeldt MF and Saris WHM (1991) Effect of different tumor types on resting energy expenditure. *Cancer Res* **51**: 6138–6141

Giacosa A, Frascio F, Sukkar SG and Roncella S (1996) Food intake and body composition in cancer cachexia. *Nutrition* **12**: S20–S23

Keller U (1993) Pathophysiology of cancer cachexia. *Support Care Cancer* **1**: 290–294

Lindmark L, Bennegard K, Eden E, Ekman L, Schersten T, Svaninger G and Lundholm K (1984) Resting energy expenditure in malnourished patients with and without cancer. *Gastroenterology* **87**: 402–408

Long NC, Morimoto A, Nakamori T and Murakami N (1992) Systemic injection of TNF-alpha attenuates fever due to IL-1β and LPS in rats. *Am J Physiol* **263**: R987–R991

Luketich JD, Rigberg D, Banchs R, Shinkwin M, Sigal R, Daly J and Mullen JL (1990) Components of energy expenditure in tumor-bearing animals. *J Surg Res* **48**: 573–578

*© 1999 Cancer Research Campaign*

McCarthy DO and Daun JM (1993) The effects of cyclooxygenase inhibitors on tumor-induced anorexia in rats. *Cancer* **71**: 486–492
McCarthy HD, McKibbin PE, Perkins AV, Linton EA and Williams G (1993) Alterations in hypothalamic NPY and CRF in anorexic tumor-bearing rats. *Am J Physiol* **264**: E638–E643
Muc-Wierzgon M, Madej K, Baranowski M and Kokot T (1996) Fluctuation of endogenous TNF alpha concentration in plasma in advanced cancer patients. *J Biol Regul Homeost Agents* **10**: 25–26
Muc-Wierzgon M, Madej K, Baranowski M and Wierzgon J (1998) Circadian rhythmometry of serum endogenous tumor necrosis factor-alpha in patients with colorectal cancer metastases. *Eur Cytokine Netw* **9**: 193–196
Ogilvie GK, Walters LM, Fettman MJ, Hand MS, Salman MD and Wheeler SL (1993) Energy expenditure in dogs with lymphoma fed two specialized diets. *Cancer* **71**: 3146–3152
Ohdo S, Koyanagi S, Yukawa E and Higuchi S (1997) Circadian rhythm of fever induced by interferon-alpha in mice. *Life Sci* **61**: 95–100
Oudart H, Heitz A, Bnouham M, Malan A and Le Maho Y (1993) Body protein and lipid deficit in tumour-bearing rats in relation to age. *Br J Cancer* **68**: 885–889
Rechcigl M, Grantham F and Greenfield RE (1961) Studies on the cachexia of tumor-bearing animals, body weight changes, carcass composition, and metabolic studies. *Cancer Res* **21**: 238–251
Roth J, Conn CA, Kluger MJ and Zeisberger E (1993) Kinetics of systemic and intrahypothalamic IL-6 and tumor necrosis factor during endotoxin fever in guinea pigs. *Am J Physiol* **265**: R653–R658
Smith BK, Conn CA and Kluger MJ (1993) Experimental cachexia – effects of MCA sarcoma in the Fischer rat. *Am J Physiol* **265**: R376–R384
Temparis S, Asensi M, Taillandier D, Aurousseau E, Larbaud D, Obled A, Bechet D, Ferrara M, Estrela JM and Attaix D (1994) Increased ATP-ubiquitin-dependent proteolysis in skeletal muscle of tumor-bearing rats. *Cancer Res* **54**: 5568–5573
Tessitore L, Costelli P and Baccino FM (1993) Humoral mediation for cachexia in tumour-bearing rats. *Br J Cancer* **67**: 15–23
Warnold I, Lundholm K and Schersten T (1978) Energy balance and body composition in cancer patients. *Cancer Res* **38**: 1801–1807

© 1999 Cancer Research Campaign
